# An Overview of the Nrf2/ARE Pathway and Its Role in Neurodegenerative Diseases

**DOI:** 10.3390/ijms22179592

**Published:** 2021-09-04

**Authors:** Emilia Zgorzynska, Barbara Dziedzic, Anna Walczewska

**Affiliations:** Department of Cell-to-Cell Communication, Medical University of Lodz, Mazowiecka 6/8, 92-215 Lodz, Poland; barbara.dziedzic@umed.lodz.pl (B.D.); anna.walczewska@umed.lodz.pl (A.W.)

**Keywords:** Nrf2, Keap1, oxidative stress, neuroprotection, neurodegenerative diseases, Alzheimer’s disease, Parkinson’s disease, Huntington’s disease, amyotrophic lateral sclerosis

## Abstract

Nrf2 is a basic region leucine-zipper transcription factor that plays a pivotal role in the coordinated gene expression of antioxidant and detoxifying enzymes, promoting cell survival in adverse environmental or defective metabolic conditions. After synthesis, Nrf2 is arrested in the cytoplasm by the Kelch-like ECH-associated protein 1 suppressor (Keap1) leading Nrf2 to ubiquitin-dependent degradation. One Nrf2 activation mechanism relies on disconnection from the Keap1 homodimer through the oxidation of cysteine at specific sites of Keap1. Free Nrf2 enters the nucleus, dimerizes with small musculoaponeurotic fibrosarcoma proteins (sMafs), and binds to the antioxidant response element (ARE) sequence of the target genes. Since oxidative stress, next to neuroinflammation and mitochondrial dysfunction, is one of the hallmarks of neurodegenerative pathologies, a molecular intervention into Nrf2/ARE signaling and the enhancement of the transcriptional activity of particular genes are targets for prevention or delaying the onset of age-related and inherited neurogenerative diseases. In this study, we review evidence for the Nrf2/ARE-driven pathway dysfunctions leading to various neurological pathologies, such as Alzheimer’s, Parkinson’s, and Huntington’s diseases, as well as amyotrophic lateral sclerosis, and the beneficial role of natural and synthetic molecules that are able to interact with Nrf2 to enhance its protective efficacy.

## 1. Introduction

Nuclear factor erythroid 2 (NF-E2)-related factor 2 (Nrf2) is conserved in the metazoan Cap ‘n’ collar (CNC) protein, belonging to the family of basic leucine zipper (bZIP) transcription factors. The CNC family of proteins comprises p45 NFE2 (nuclear factor erythroid-derived 2), three NF-E2-related factors (Nrf1, Nrf2, and Nrf3), and two BTB and CNC homology (Bach) proteins, Bach1 and Bach2 [1]. The CNC transcription factors form heterodimers with small musculoaponeurotic fibrosarcoma proteins (sMafs) that either activate or repress the transcription of target genes. The heterodimer Nrf2/sMaf orchestrates the transcription of proteins that collectively favor cell survival through binding to the antioxidant response element (ARE, 5′-(A/G)TGACNNNGC(A/G)-3′), a *cis*-acting element of DNA, also known as the electrophile response element (EpRE) [2,3,4,5]. Nrf2 not only responds to environmental stressors by the activation of numerous antioxidant, detoxifying and cytoprotective enzymes and restoring cellular homeostasis, but also contributes to constitutive gene expression, as evidenced by cell-transfection and in vivo studies on Nrf2-/-mice [6,7,8,9]. Dysfunctions in the Nrf2/ARE signaling pathway result in a loss of redox homeostasis, leading to the overloading with reactive oxygen/nitrogen species (ROS/RNS) that is a common pathological hallmark of neurodegenerative diseases [10]. Furthermore, increasing evidence indicates that Nrf2 also plays a role in many other cellular activities, such as DNA repair, autophagy, intermediary metabolism and mitochondrial function (for a review, see [11]).

This review discusses the molecular regulation of Nrf2 activity and the contribution of the Nrf2/ARE-driven transcriptional program in most common neurodegenerative diseases. It also examines the potential of Nrf2 activation as a therapeutic target in the treatment of widespread neurological diseases associated with an aging population.

## 2. Regulation of Nrf2 Transcription

Nrf2 is encoded by the nuclear factor (erythroid-derived 2)-like 2 gene (*NFE2L2*) located in the cytogenetic band 2q31.2 of chromosome 2 in humans (gene ID: 4780). The *NFE2L2* consists of five exons interrupted by four introns [12] giving a rise of 8 transcript variants encoding 6 isoforms of Nrf2 (Table 1) [13,14,15]. In the *NFE2L2* promoter are two ARE-like sequences, through which Nrf2 can enhance its own expression [16], as well as the xenobiotic response element (XRE)-like elements binding the aryl hydrocarbon receptor (AhR) transcription factor that is a transducer of environmental pollutants in eukaryotic cells [17,18]. The promoter of *NFE2L2* does not contain TATA and CCAAT boxes, the common promoter elements [19], but it is rich in CpG islands, clusters of CpG dinucleotides whose methylation silences gene transcription [20], hence, *NFE2L2* is under a strong epigenetic regulation (for a review, see [21]). At the upstream of the start sequence in *NFE2L2*, the κB2 region for NF-κB binding [22] is present, which allows direct stimulation of the Nrf2 expression in the course of acute inflammation, or tumorigenesis by p65 NF-κB [23]. Furthermore, several binding sites for the activating enhancer-binding protein 2 (AP-2) transcription factor were found in the *NFE2L2* promoter [19], which may interact with a range of proteins that may function as a co-activator or suppressor of gene transcription [24].

Chromatin immunoprecipitation-sequencing (ChIP-seq) data indicates the existence of Myc and Jun binding sites at the transcriptional start site in exon1 of *NFE2L2* [25]. The proto-oncogenes c-Myc and c-Jun, involved in cell cycle progression and cellular proliferation, are frequently upregulated in human tumors [26,27]; thus, they enhance Nrf2-dependent anticancer drug resistance [28]. Similarly, the oncogene KRAS may bind to the 12-O-tetradecanoylphorbol-13-acetate (TPA)-response element (TRE) present in the *NFE2L2* and reduce the sensitivity of cancer cells to chemotherapeutics [29]. Nrf2 expression may also be upregulated by the breast cancer type 1 susceptibility protein (BRCA1), which functions as a co-activator for the nuclear translocator/aryl hydrocarbon receptor (ARNT/AhR) heterodimer interacting with the XRE-like sequence in the *NFE2L2* [30], or it can act as a direct activator of *NFE2L2* [31]. Regarding the mechanisms of Nrf2-dependent cytostatic drug resistance, *NFE2L2* expression is upregulated also by the ubiquitously expressed specificity protein 1 (Sp1), a nuclear transcription factor belonging to the C2H2-type zinc-finger protein family. However, this regulation not only increases cancer drug resistance [32] but also prevents oxidative stress-induced DNA damage and the death of cortical neurons [33].

Numerous studies have demonstrated that Nrf2 mRNA transcripts are negatively regulated by microRNAs (miRNAs) [34,35,36,37]. These short, single-stranded non-coding RNAs regulate gene expression, either by an enhancement of a target mRNA degradation or inhibition of the translation in a sequence-specific manner [38]. The first miRNA identified as a negative regulator of Nrf2 was miR-144. It was found that miR-144 reduces Nrf2 levels in pathological red blood cells, interacting with two evolutionarily conserved target sites in the 3′ UTR of *NFE2L2* (at 265–271 bp and 370–377 bp) [39]. Following this, miR-28 was found to negatively regulate Nrf2 expression in breast epithelial cells [40]. Since then, evolutionarily conserved binding sites for other miRNAs (miR-142-5p, miR-153 and miR-237) have been identified in human *NFE2L2* that directly downregulated Nrf2 levels [41]. It should be noted, however, that miRNA may also facilitate the activation of Nrf2 to promote cell survival under stress conditions. Recent studies on cells subjected to oxygen and glucose deprivation/reperfusion (OGD/R) indicate that miRNA-380-5p facilitates the activation of the Nrf2-Keap1 signaling pathway through direct repression of Bach1, a competitor for Nrf2 for binding to the ARE site in DNA [42]. Furthermore, miR-152-3p overexpression alleviates OGD/R-induced neuronal injury by reinforcing Nrf2/ARE signaling via direct inhibition of postsynaptic density protein 93 (PSD-93), an activator of Fyn kinase responsible for Nrf2 degradation [43].

## 3. Regulation of Nrf2 Activity

Under normal conditions, Nrf2 is a short-lived protein that is continuously ubiquitylated and targeted to proteasomal degradation. Following oxidative or electrophilic insult, Nrf2 is activated by complex and tightly regulated mechanisms. In the modulation of Nrf2 activity, next to the regulation of Nrf2 transcription described above, numerous interactions of Nrf2 are involved with its inhibitor Keap1 (Kelch-like ECH-associated protein 1), and the interaction of Keap1 with other proteins. Furthermore, numerous kinases, through the phosphorylation of amino acid residues in Nrf2 and Keap1, determine the fate of Nrf2 in the cell, ubiquitination, or sharing transcriptional co-activators for the ARE-driven gene network.

### 3.1. Structure of Nrf2 and Keap1 Inhibitor

**Figure 1:**
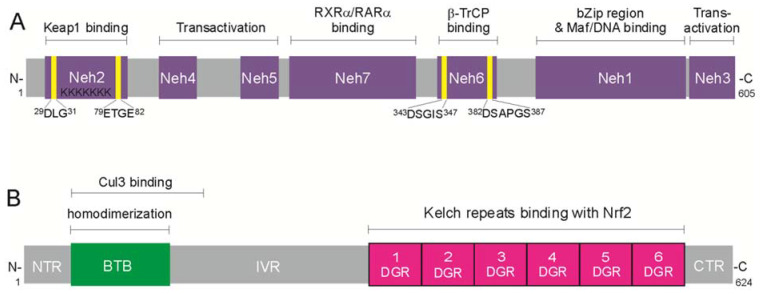
Multi-domain organization of Nrf2 transcription factor and Kelch-like ECH-associated protein 1 (Keap1)—the repressor of Nrf2. (**A**) A 605-amino acid Nrf2 contains seven functional domains. The N-terminal Neh2 has two ^29^DLG^31^ and ^79^ETGE^82^ motifs that bind the Keap1 homodimer, which suppresses Nrf2 and mediates its ubiquitin-dependent proteasomal degradation; the Neh4 and Neh5 recruit transcriptional co-activators, CREB-binding protein (CBP), and/or repressor-associated coactivator (RAC); the Neh7 domain mediates repression of Nrf2 by retinoid X (RXR) and retinoic acid (RAR) receptors; the Neh6 has two ^343^DSGIS^347^ and ^382^DSAPGS^387^ motifs interacting with β-transducin repeat-containing protein (β-TrCP) and is responsible for the β-TrCP-mediated proteasomal degradation; the Neh1 contains a basic region-leucine zipper motif and is responsible for dimerization with small musculoaponeurotic fibrosarcoma (Maf), or BTB and CNC homology (Bach) proteins, the heterodimeric partners for Nrf2 to recognize the ARE sequence in target gene promoters; the C-terminal Neh3 domain is a transactivation domain that recruits chromodomain helicase DNA-binding domain protein 6 (CHD6). (**B**) Keap1 protein, the repressor of Nrf2, comprises five functional domains: the NTR domain in N-terminal region; the BTB domain, essential for homodimerization and for binding with Cul3-Rbx1 ligase complex; the intervening region (IVR), containing cysteine residues sensitive to oxidation; the double-glycine repeats (DGR)/Kelch domain, containing six Kelch-repeats, which operates as the binding sites for Nrf2; and the C-terminal region (CTR).

Nrf2 is a 605 amino acid protein that has seven functional domains called Neh (N2-erythroid-derived Cap ’n’ Collar homology) domains presented in Figure 1A. The Neh1 domain contains a basic region-leucine zipper motif binding as well as the sequence for heterodimerization with small Maf transcription co-activators. The Neh2 domain possesses two motifs, ^29^DLG^31^ and ^79^ETGE^82^, through which Nrf2 binds to the repressor, the Keap1 homodimer that promotes Nrf2 ubiquitination and 26S proteasomal degradation. The third DIDLID (17–32 aa) motif in the Neh2 domain is indispensable for Neh2 activity and appears to be necessary to recruit a ubiquitin ligase to the protein [44]. In the C-terminal, the Neh3 domain is a transcriptional co-activator that recruits chromodomain helicase DNA-binding domain protein 6 (CHD6) [45]. The Neh4 and Neh5 domains act synergistically to recruit transcriptional co-activators, the CREB-binding protein (CBP), and/or repressor-associated coactivator (RAC) [46]. The Neh6 domain contains two motifs, DSGIS and DSAPGS, degrons for Keap1-independent Nrf2 degradation through the β-transducin repeat-containing protein (β-TrCP), creating a complex with the S-phase kinase-associated protein 1 (Skip1)-Cul1-Rbx1 E3 ubiquitin ligase [17]. Finally, the Neh7 domain mediates the repression of Nrf2 by retinoid X (RXRs) and retinoic acid (RARs) receptors, which prevent the binding of the transcription co-activators to the Neh4 and Neh5 domains [47].

In the cytoplasm, the half-life of Nrf2 is less than 20 min [48,49], due to its being bound by the plentiful inhibitor Keap1. Keap1 is a 624-amino acid member of the Kelch-like family of proteins containing the BTB (broad complex/tram track/bric-a-brac) domain. This multi-domain protein is composed of (1) the N-terminal region (NTR), (2) the BTB region essential for forming the Keapl homodimer and for the binding of a Cul3-Rbx1 ligase (CRL) complex, (3) an intervening region (IVR) that is rich with cysteines, also named the BTB and C-terminal Kelch (BACK) domain, (4) a double-glycine repeat (DGR) containing six Kelch repeat motifs which form Nrf2 binding sites, and (5) the C-terminal region (CTR) (Figure 1B).

### 3.2. Keap1-Dependent Nrf2 Activation

In the cytoplasm of non-stressed cells, Nrf2 is sequestered by a Keapl homodimer that complexes the Cul3-Rbx1 E3 ubiquitin ligase, thus promoting Nrf2 polyubiquitination and continual proteasomal degradation. The presence of increased levels of oxidative or electrophilic compounds changes the conformation of Keap1 and allows Nrf2 activation [22]. The mechanism by which Nrf2 disconnects from Keap1 remains unclear. In the “hinge and latch” model, through its BTB domain, Keap1 creates a homodimer that binds with two motifs in the Neh2 domain of Nrf2 through Kelch domains. The high-affinity ETGE motif binds one Keap1 molecule as a “hinge”, and a low-affinity DLG motif functions as a “latch” for another Keapl molecule. The oxidative/electrophilic modification of cysteines (C273 and C288) in the IVR domain of the Keap1 dimer induces a conformational change of Keap1 that results in its dissociation from the DLG motif (the hinge) and disrupts the BTB-Cul3 ligase interaction required for Nrf2 ubiquitination. According to this “hinge and latch” model, the stoichiometric ratio of Keap1 to Nrf2 in the complex should be 2:1; however, studies did not confirm this ratio [50]. Therefore, the “quaternary model” was proposed. In this model, the Kelch domain of one Keap1 binds Nrf2 through the ETGE motif, and the other molecule of the dimer binds through the ESGE motif to phosphoglycerate mutase 5 (PGAM5), anchored in the outer mitochondrial membrane [51]. In this model, under stress conditions, Nrf2 degradation is impaired, but not the interaction with the high-affinity motif. However, later stoichiometry measurements supported a 2:1 ratio of Keap1 and Nrf2 in the complex [52]. Nrf2 may be more accessible for nuclear import due to blocking the binding site with Nrf2 through Keap1-protein interaction. Among the proteins containing Keap1-interacting region (KIR)-like ETGE motifs, which compete with the Nrf2 in binding to Keap1, are p62/sequestosome 1 (SQSTM1), dipeptidyl peptidase 3 (DPP3), the Wilms tumor gene on the X chromosome (WTX) and a partner and localizer of BRCA2 (PALB2) [53]. It was also found that the p21 protein competes with Keap1 for Nrf2 binding through the DLG motif [54].

### 3.3. Keap1-Independent Nrf2 Activation

Three E3 ubiquitin ligase complexes, (1) βTrCP-S-phase kinase-associated protein-1 (Skp1)-Cul1-Rbx1, (2) HMG-CoA reductase degradation 1 (Hrd1) and (3) WD-repeat protein 23 (WDR23)-Cul4-damaged DNA binding protein 1 (DDB1), are known to be involved in Keap1-independent Nrf2 degradation.

Beta-TrCP binds to DSGIS and DSAPGS degrons in Neh6 domain of Nrf2 through ~40 amino acid motifs, having Trp-Asp (W-D) dipeptide repeats (WD40) and forms the ubiquitin ligase complex with Skp1, Cul1, and Rbx1 proteins. Phosphorylation of the DSGIS motif in Nrf2 by GSK-3 enhances the binding of β-TrCP to Nrf2 [55] and Nrf2 degradation. It is possible that the phosphatase and TENsin homology protein (PTEN) augments GSK-3-mediated phosphorylation of the Nrf2 domain and Keap1-independent Nrf2 degradation [56]. The inhibition of GSK-3 activity via phosphorylation of its N-terminal residues by protein kinase B/ serine/threonine-protein kinase (PKB/Akt), p38 mitogen-activated protein kinase (p38 MAPK) or protein kinase C (PKC) increases the availability of Nrf2 for the transcriptional machinery of ARE-driven genes [57]. Following activation, GSK-3 increases the nuclear accumulation of Fyn kinase, the Src-A kinase family member, by which phosphorylating tyrosine residue in the Neh1 domain stimulates the nuclear export, ubiquitination, and degradation of Nrf2 [58,59]. A cell-based ubiquitylation analysis revealed another ubiquitin ligase, Hrd1 that, independently from Keap1 and β-TrCP, attenuates Nrf2 signaling by binding to Neh4–Neh5 domains. It is important to note that the Hrd1-mediated ubiquitylation and degradation of Nrf2 have only been reported in the cytosol [60]. The ubiquitination and degradation of Nrf2 are also controlled by WDR23, a WD40-repeat protein that binds the DIDLID sequence in the Neh2 domain and interacts with the Cul4-DDB1 complex [61]. Since WDR23 is also localized in the nucleus [62], it is possible that post-translational regulation of Nrf2 activity may not be limited to the cytoplasm.

In addition, Nrf2 activity is regulated by kinases that directly phosphorylate the Nrf2 molecule. The phosphorylation of Ser-40 in the Neh2 domain by PKC inhibits complex formation with Keap1 and facilitates the nuclear import of Nrf2 [63]. The phosphorylation of serine and threonine in the Neh4 and Neh5 domains by casein kinase 2 (CK-2) increases the nuclear Nrf2 level and transcriptional activity [64]. Furthermore, the phosphorylation of serine and threonine residues (S215, S408, S558, T559 and S577) in Neh1, Neh3 and Neh7 domains by MAPKs also enhances Nrf2 nuclear accumulation [46]. Another possible mechanism of Nrf2 activation is regulated by PI3K. In an oxidative environment, activated PI3K depolymerizes actin, which tethers the Nrf2-Keap1 complex and allows Nrf2 to escape from Keap1. Recently, it has been demonstrated that the synergy between Keap1/Nrf2 and phosphoinositide 3-kinase (PI3K) signaling pathways leads to certain types of carcinoma [65]. Moreover, Nrf2 translocation to the nucleus is regulated by 5′ AMP-activated protein kinase (AMPK) activity; thus, its activation is strictly dependent on cellular energy levels [66].

**Figure 2:**
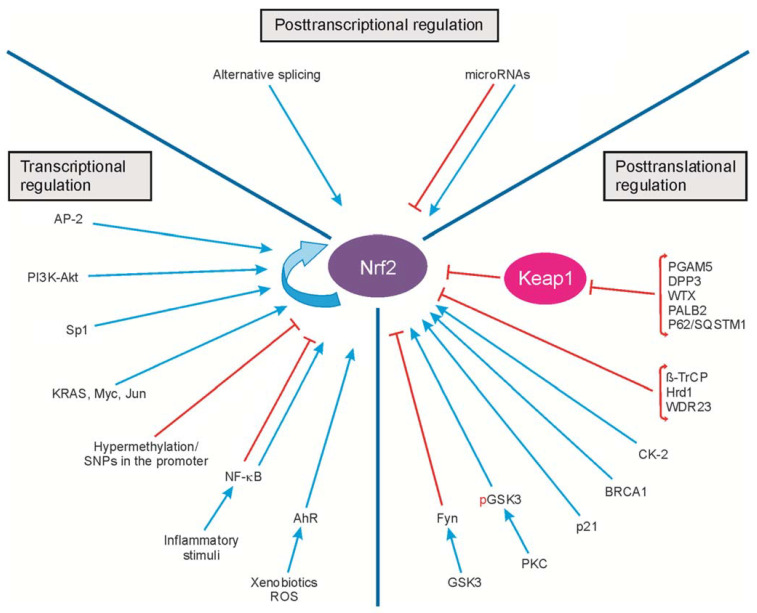
Transcriptional, posttranscriptional and posttranslational regulation of Nrf2 activity. The transcription of Nr2 is regulated by the transcription factors, oncogenes (KRAS, Myc, Jun) and modifications of the Nrf2 promoter (hypermethylation or SNPs). Posttranscriptional regulation includes alternative splicing and microRNA binding. The posttranslational control of Nrf2 activity results from protein modifications, protein degradation and protein–protein interactions. Arrows and blunt ends indicate activation and inhibition, respectively. ROS, reactive oxygen species; SNPs, single nucleotide polymorphisms; Akt, serine/threonine-protein kinase; BRCA1, breast cancer susceptibility 1; PI3K, phosphoinositide 3-kinase; NF-κB, nuclear factor-κB; AhR, aryl hydrocarbon receptor; Fyn, tyrosine-protein kinase Fyn; GSK-3, glycogen synthase kinase-3; pGSK-3, phosphorylated glycogen synthase kinase-3; KRAS, GTPase KRas; Myc, Myc proto-oncogene protein; Jun, transcription factor AP-1; AP-2, activating enhancer-binding protein 2; Sp1, specificity protein 1; PKC, protein kinase C; CK-2, casein kinase 2; β-TrCP, β-transducin repeat-containing protein; Hrd1, HMG-CoA reductase degradation 1; WDR23, WD-repeat protein 23; PGAM5, phosphoglycerate mutase 5; DPP3, dipeptidyl peptidase 3; WTX, Wilms tumor gene on the X chromosome; PALB2, partner and localizer of BRCA2; p21, cyclin-dependent kinase inhibitor 1; p62/SQSTM1, sequestosome 1. Figure adapted from [67].

The transcriptional, posttranscriptional and posttranslational regulation of Nrf2 activity is presented in Figure 2.

## 4. Nrf2-Signaling for ARE-Driven Genes

Nrf2 is a master transcription factor that supports cellular defense against exogenous electrophilic/cytotoxic agents and harmful endogenous processes, by enhancing the expression of detoxifying enzymes, maintaining redox homeostasis, and coordination of the cell cycle and intermediary metabolism. Nrf2/ARE transcriptional regulation coordinately modulates the activity of genes that collectively favor cell survival.

For gene transcription, Nrf2 requires various binding partners, such as co-activators and chromatin remodelers. Of these, small Mafs (MafK, MafG, and MafF) are obligatory co-activator dimeric partners that bind to the Neh1 domain through their leucine zipper domains, thus allowing them to recognize the ARE sequence of DNA in the target gene promoter region [2]. Nrf2 also dimerizes with Bach proteins, which prevent Nrf2 from binding to ARE sites. As such, the Nrf2-Bach heterodimer complex inhibits the transcription of Nrf2-dependent genes. Such downregulation has been demonstrated for heme oxygenase-1 (HO-1) [68,69], NAD(P)H:quinone dehydrogenase 1 (NQO1) [70] and immunoglobulin heavy chain gene 3` enhancer [71].

To make the chromatin structure accessible to the RNA polymerase II and to initiate transcription, Nrf2 recruits co-activators and components of the transcription machinery through protein–protein interactions. It has been demonstrated that CBP/p300, which possesses histone acetyltransferase activity through its cysteine–histidine-rich region 3 (CH3) domains, binds to the Neh4 and Neh5 domains of Nrf2; this results in chromatin remodeling and the activation of gene transcription via ARE sequences [46,72,73]. However, it has been demonstrated that Nrf2 and NF-κB compete for binding with CBP/p300, which facilitates the recruitment of histone deacetylase 3 to MafK [74]; therefore, in case of the aggravation of inflammation, the Nrf2/ARE pathway can be suppressed by competition for the same transcriptional cofactors in the nucleus [75]. Another transcriptional co-activator of Nrf2 is CHD6, which may be involved in recruiting components of the transcriptional complex and interacts with the VFLVPK motif in the Neh3 domain [45].

In addition to histone-modifying enzymes, Nrf2 interacts with the mediator, an evolutionarily conserved multiprotein complex essential for RNA polymerase II-driven transcription. Sekine et al. [76] report that the mediator complex subunit 16 directly activates Nrf2 transcription and supports the electrophile-induced transcription of Nrf2-dependent target genes via either the Neh4/Neh5 or Neh1 domains. Moreover, Nrf2 can regulate transcription by cooperating with other transcription factors: Jun-D, c-Jun, and Jun-B [77], activating transcription factors 3 and 4 (ATF3, ATF4) [78,79], runt-related transcription factor 2 (Runx2) [80], and Yin Yang 1 (YY1) [81]. These participate in the maintenance of cell homeostasis in the brain and may cooperate with replication protein A1, a single-strand DNA-binding protein essential for DNA replication, repair and recombination [82].

**Figure 3:**
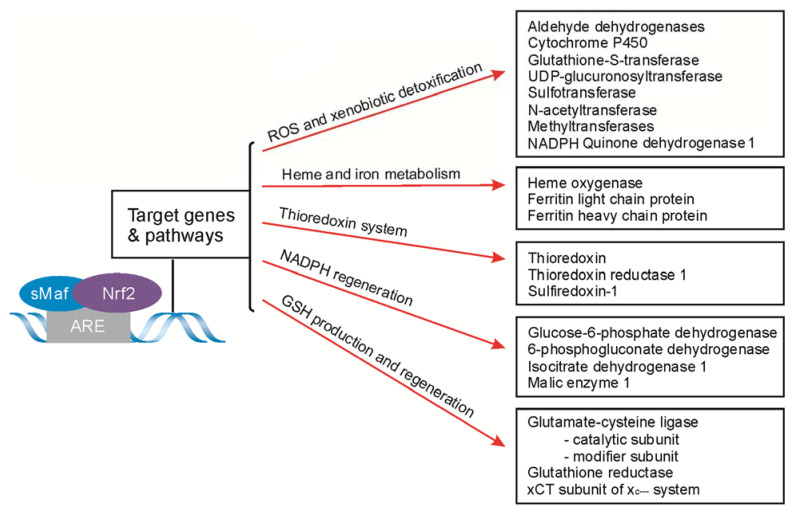
The principal cytoprotective enzymes encoded by the ARE-driven genes. GSH, glutathione; NADPH, reduced nicotinamide adenine dinucleotide phosphate; UDP, uridine diphosphate; xCT, cystine/glutamate transporter.

The Nrf2/ARE pathway controls dozens of genes of enzymes participating in fundamental cell-rescue mechanisms. In relation to the specificity of their action, they can be classified into five categories (Figure 3). The first group of Nrf2/ARE-dependent genes encodes the proteins involved in ROS and xenobiotic detoxification. This group includes a wide range of enzymes starting from phase 1 detoxification, such as the family of aldehyde dehydrogenases, NQO1 and cytochrome P540, through the enzymes of phase 2 conjunctive enzymes (glutathione S-transferase (GST), UDP-glucuronosyltransferase (UGT), sulfotransferases, N-acetyltransferases, and methyltransferases) [83], ending with the transporters of the ATP-binding cassette protein superfamily (ABC) taking part in excretion, or the import of various molecules across the cell membranes [84]. The second cluster of genes controlled by the Nrf2/ARE pathway is the group for GSH-produced and GSH-regenerated enzymes that control cellular redox homeostasis. These include rate-limiting in the synthesis of GSH glutamate-cysteine ligase subunits: the catalytic subunit (GCLC) and modifier subunit (GCLM), glutathione reductase (GS), and the cystine/glutamate transporter (xCT) subunit of the x_c_^−^ system that supply cysteine to cells for GSH synthesis [85,86]. The third group consists of genes encoding the proteins responsible for heme and iron metabolism. To this group belongs HO-1, catalyzing heme molecule breakdown, and subunits of the ferritin complex, responsible for iron transport and storage: a ferritin light-chain protein encoded by the *FTL* gene, and a ferritin heavy polypeptide that is a ferroxidase enzyme encoded by the *FTH1* gene. The other Nrf2/Maf dimer target genes are those for NADPH-regenerated enzymes, which include glucose-6-phosphate dehydrogenase (G6pd), 6-phosphogluconate dehydrogenase (Pgd), isocitrate dehydrogenase 1 (Idh1) and malic enzyme 1 (Me1) [87]. The last group of Nrf2-dependent genes is those encoding the thioredoxin (TXN)-based antioxidant system, composed of TXN, thioredoxin reductase 1 (TrxR), and sulfiredoxin 1 (Srx1), that maintain protein thiols in a reduced state [88]. Finally, Nrf2 has the ability to induce the expression of diverse genes with cytoprotective activity; thus, it plays a pivotal role in cell response to various stimuli, allowing cellular adaptation and maintaining homeostasis.

Cumulative evidence indicates that, among the Nrf2-regulated genes, there are also those involved in functions other than redox homeostasis. The Nrf2/ARE pathway supports DNA repair after cell radiation, irrespective of ROS levels, since inhibition of Nrf2 by all-trans retinoic acid and Nrf2 knockdown results in more severe DNA damage [89] and increases DNA double-strand breaks [90]. Hence, an increase in Nrf2 protein levels results in the activation of PI3K-related regulators of the DNA damage response and G2 cell cycle arrest, with the aim of maintaining genome integrity [90]. Nrf2 is also recognized as the transcription factor regulating autophagy gene expression, which can play an important role in the clearance of neurotoxic protein aggregates in Alzheimer’s disease [91]. Furthermore, it has been demonstrated that Nrf2 plays a prominent role in supporting mitochondrial function, particularly in stress conditions, through the upregulation of the transcription of uncoupling protein 3 [92] and mitochondrial genes, such as the gene-encoding translocase of the outer mitochondrial membrane 70 (TOMM70). In addition, Nrf2 plays a key role in mitochondrial biogenesis [93], interacting with the peroxisome proliferator-activated receptor-γ coactivator-1α (PGC-1α), the principal mitochondrial DNA transcription coactivator [94].

## 5. Role of the Nrf2/ARE Pathway in Neurodegenerative Diseases and Potential Therapeutic Targets

The prevalence of neurological diseases, including dementia and motor disorders caused by neurodegeneration, is expected to increase with a longer life expectancy. Although oxidative stress has been shown to be involved in various neurodegenerative diseases [95], the cytopathological role of Nrf2 is multi-directional and multi-level, so that numerous potential targets in Nrf2 activation and Nrf2-driven gene transcription can be applied for the delay in onset of neurodegenerative diseases and even in the therapy of brain disorders.

### 5.1. Alzheimer’s Disease

Neurodegenerative diseases offer a broad spectrum of neurological diseases. Most often they are caused by the accumulation of intraneuronal bundles of hyperphosphorylated tau protein, in the form of neurofibrillary tangles, neuritic plaques and dystrophic neurites [96], as well as extracellular aggregates of beta-amyloid plaques, all characterizing Alzheimer’s disease (AD). However, the link between amyloid deposits and neurofibrillary tangles is not yet clear, despite at least three decades of research devoted to finding the trigger factors of these proteinopathies and to understanding the timeline of molecular and biochemical events leading to their pathology [97]. To date, views on the amyloid or tau pathologies in AD compete with each other (arguments for “the amyloid hypothesis”, see [98], for “tau axis hypothesis”, see [99]). Notwithstanding, whichever proteinopathy appears first, in both cases, oxidative stress can be an essential factor in their triggering. Therefore a failure of the Nrf2/ARE signaling pathway responsible for redox/electrophilic homeostasis can change the enzymatic machinery, leading to sequential amyloidogenic cleavage of the amyloid precursor protein (APP) [100] and an increase in tau phosphorylation by activated GSK-3β, cyclin-dependent-like kinase 5 (Cdk5), and multiple other kinases [101]. Indeed, in the brain [102,103], as well as in the urine and blood plasma [104] of AD patients, a significant reduction of antioxidant enzyme activity and increased levels of oxidative stress markers, such as 4-hydroxynonenal (4HNE), thiobarbituric acid reactive substances (TBARs), have been demonstrated. In accordance with studies of AD-affected brains, pre-clinical studies on Nrf2-knockout transgenic mice with amyloidopathy and tauopathy reported that an Nrf2 deficit was associated with increased markers of oxidative stress and neuroinflammation, compared to the control mice. Moreover, Nrf2 deficiency led to an increased accumulation of insoluble phosphorylated tau oligomers and amyloid deposits in the hippocampus, which was clinically manifested as impaired cognition function [105,106]. Contrastingly, increased NQO1 activity and HO-1 levels were reported in neurons and astrocytes isolated from the post-mortem brains of AD patients [107,108]. It should be considered, however, whether these Nrf2-driven enzymes increased during the lifetime of the patients or arose as the effect of hypoxia after cardiac arrest.

Some results indicate that Nrf2 signaling may not respond properly to oxidative stress in an AD brain. Immunochemistry and immunoblotting studies of AD post-mortem brains have shown the predominantly cytoplasmic, and not nuclear, localization of Nrf2 in the hippocampal neurons and astrocytes, compared to age-mated controls [109]. This suggests the inefficient expression of Nrf2-driven cell rescue genes, despite marked signs of oxidative damage to neurons, however specifically in AD, because the same study revealed the nuclear localization of Nrf2 in the brain of Parkinson’s diseasse patients. In accordance with this finding, based on analysis of genetic variation in *NFE2L2* and *KEAP1* in material from hundreds of AD patients, von Otter et al. [110] demonstrated no association between the single nucleotide polymorphisms (SNPs) or haplotypes of Nrf2 and AD risk and suggested that amyloid β (Aβ) aggregates impair the Nrf2 system, not the opposite. However, they have found a faster progression of AD associated with the GAAAA Nrf2 haplotype.

The pathogenesis of AD is linked to mitochondria dysfunction and defective mitochondrion dynamics [111], as well as to enhanced mitophagy [112]. Some of these effects are evoked by APP and Aβ interaction with mitochondrial proteins [113]. In accordance with these findings, the over-expression of APP resulted in the downregulation of Nrf2 levels among numerous other proteins participating in mitochondrial fusion, biogenesis and mitophagy [114]. Furthermore, the inactivation of Nrf2 in primary neuron culture impaired the activity of the electron transport chain complexes, decreasing reducing molecules (NADH, FADH2) levels and the efficacy of oxidative phosphorylation [115]. Therefore, the health of mitochondria is strictly dependent on Nrf2/ARE pathway signaling, which likely does not work properly in the progress of tauopathy and amyloidogenesis in AD patients.

Notwithstanding these points, in vivo and in vitro studies have demonstrated that the pharmacological activation of the Nrf2/ARE pathway protects against Aβ toxicity. Treatment with a potent inducer of Nrf2, tert-butylhydroquinone, or transfection with the Nrf2 gene, increased the cell viability of neurons in vitro and reduced Aβ formation and ROS generation in double transgenic mice expressing the amyloid-β precursor protein and mutant human presenilin 1 (*APP*/*PS1*) [116,117,118]. Moreover, the induction of Nrf2 shifted the balance from the soluble to the insoluble Aβ fraction, thus possibly reducing the levels of toxic soluble Aβ peptides [119]. Some evidence indicates that Nrf2 may reduce tau accumulation and induce ARE-driven gene transcription, by upregulation of the nuclear dot protein 52 (NDP52) and p62/sequestosome1 (SQSTM1), an autophagy adaptor protein involved in the proteasomal degradation of tau and Keap1 [91,120,121].

An effective strategy to combat oxidative stress is supplementation with cytoprotective and antioxidant phytochemicals that are capable of relieving neurological symptoms or even delaying their onset. Numerous preclinical and clinical studies in AD patients indicate that various phytochemicals, including naringenin, curcumin, methysticin, resveratrol, berberine, trigonelline, astaxanthin and sulforaphane, may have beneficial effects, in part, by enhancing Nrf2/Keap1/ARE pathway activity. Among these, resveratrol yielded favorable results in reducing Aβ1-42-induced toxicity in a neuronal cell line [122], and in decreasing the density of Aβ plaques in the cortex and hippocampus of mice [123]. In addition, resveratrol supplementation in a mouse AD model had neuroprotective and pro-survival effects, decreasing the amyloid burden and reducing tau hyperphosphorylation [124]. Curcumin is a supplement that is frequently studied regarding the alleviation of AD symptoms and is the main component of turmeric (*Curcuma longa*) extract. However, the results of clinical trials with curcumin and resveratrol supplementation are not consistent. Short-term treatment with curcumin [125] was found to have a beneficial effect on working memory and sustained attention in healthy elderly patients, but not in AD patients [126]. Similarly, a randomized, placebo-controlled, double-blind trial demonstrated that one-year dietary supplementation with resveratrol slowed the progression of cognitive and functional decline in mild to moderate AD subjects [127], while Zhu et al. demonstrated no significant changes in the cognitive abilities and mental state between the experimental and placebo groups [128]. These discrepancies may result from the distinct bioavailability of the phytochemicals used in these studies [129,130], but also may depend on the stage and symptom severity of AD.

### 5.2. Parkinson’s Disease

The second most common neurodegenerative disorder after Alzheimer’s disease is Parkinson’s disease (PD), which is characterized by a loss of dopaminergic neurons in the substantia nigra and the accumulation in the neurons of α-synuclein (ASN) oligomers, known as Lewy bodies. Approximately 10% of patients have inherited early-onset PD, which is associated with a mutation of the *SNCA* gene encoding ASN, and a mutation of *PRKN* gene encoding the E3 ubiquitin-protein ligase parkin, which is essential for the ubiquitin-proteasome system [131]. However, the vast majority of PD cases have an idiopathic cause for ASN oligomerization, leading to the destruction of dopamine nerve terminals projecting to the striatum. Among the etiological factors of sporadic PD, next to mitochondrial dysfunction and neuroinflammation, oxidative stress and the impairment of the Nrf2/ARE pathway have been implicated [132]. Many studies indicate an increase in the markers of oxidative damage, along with decreased antioxidant enzyme activity, in the brain and blood of PD patients [133,134,135,136]. In addition, decreased Nrf2 activity [137], a loss of the dopaminergic neurons after Nrf2 deletion that are associated with microglia activation [138], and the exacerbation of synuclein aggregation [139] in mouse models of PD have been demonstrated. On the other hand, a meta-analysis of nine PD microarray datasets revealed the increased levels of Nrf2 associated with the downregulation of 31 genes containing the ARE consensus sequence [140]. Although results concerning Nrf2 expression in PD are inconclusive, impaired Nrf2 signaling is likely to be insufficient to overcome the oxidative damage of cells. Indeed, the activation of Nrf2 has been associated with neuroprotection in various PD models. Keap1 knockdown by short interfering RNA (siRNA) resulted in the increased expression of Nrf2/ARE-driven genes that protect against oxidative stress in astrocytes. Moreover, these Keap1-silenced astrocytes modestly protected dopaminergic neuron terminals against the toxicity of 1-methyl-4-phenyl-1,2,3,6-tetrahydropyridine (MPTP) injected into the striatum [141]. Likewise, in the cellular model of ASN aggregation, the Nrf2/ARE-dependent upregulation of HO-1 was found to protect cells against ferrous ion-induced toxicity [142].

The activation of Nrf2 by various natural and synthetic compounds is a potential therapeutic target for PD. Lou et al. [143] reported that the bioflavonoid naringenin, present in grapefruit, demonstrates the Nrf2-dependent neuroprotective effect. It was found that naringenin also protects against 6-hydroxydopamine (6-OHDA) toxicity by increasing the Nrf2, HO-1, GCL and GSH levels in in vitro and in vivo models of PD. Another natural compound, Schisandrin B, isolated from *Schisandra chinensis*, a herb used in traditional Chinese medicine, has been demonstrated to protect neuroblastoma cells from 6-OHDA-induced toxicity and 6-OHDA-induced neuron destruction in vivo. Moreover, Schisandrin B inhibited microR-34a expression and increased the expression of Nrf2/ARE-dependent HO-1 and NQO1, suggesting that it inhibits the negative regulatory mechanism of miR-34a on the Nrf2 pathway [144]. Increasing evidence has been shown that sulforaphane (SFN) may become a candidate for the treatment of PD. In MPTP-treated mice, SFN induced the expression of antioxidant enzymes, reduced ROS levels, increased mitochondrial biogenesis, and prevented dopaminergic neuronal loss [145]. In cells treated with SFN, it suppressed GSK-3β activity and modulated Keap1 cysteines in the BTB domain, IVR, Kelch repeat domain and C-terminal domain, and disrupted Nrf2-Keap1 binding, thus allowing the nuclear accumulation of Nrf2 [146,147]. Moreover, virtual screening of the Asinx and ChemDiv database by Kim and coworkers resulted in the identification of a small-molecule Nrf2 activator, KKPA4026. This compound induced Nrf2-dependent antioxidant enzymes, HO-1, GCL and NQO1, effectively attenuated PD-associated behavioral deficits and protected dopaminergic neurons in a mouse model of PD [148]. In addition, novel curcumin-based analogs (diethyl fumarate hybrids), acting as dual modulators of both GSK-3β inhibition and Nrf2 induction, were designed for PD treatment [149]. The hybrids showed a marked ability to activate Nrf2 and increase neuronal resistance to oxidative stress, as well as showing neuroprotective effects against ASN and 6-OHDA toxicity. Actually, the number of natural and synthetic Nrf2 activators that may have an impact on PD pathology is still increasing.

### 5.3. Huntington’s Disease

Huntington’s disease (HD), an autosomal dominant neurodegenerative disease, is caused by an accumulation of the trinucleotide CAG repeats within the *HTT* gene, resulting in an expansion of polyglutamine repeats in the huntingtin protein (mutant huntingtin, mtHtt). Clinically, HD is manifested by motor impairment, including the loss of movement coordination, as well as various cognitive and psychiatric disturbances. Although the etiology of the disease is complex, evidence suggests that mitochondrial dysfunction and the failure of the cellular antioxidant system play a key role in HD pathology. In the brains of HD patients [150], as well as in the brain and plasma of HD transgenic mice [151,152,153,154], enhanced lipid peroxidation, correlated with disease progression [152,154] and severity [151], was demonstrated. The insufficiency of the antioxidant mechanism, in part, may result from the inhibition of Nrf2 activity by mtHtt, which directly interacts with the CBP/p300 dimer and inhibits its acetylase activity [155,156], and is essential for Nrf2 stability and cellular localization [157].

Although Nrf2 activation was found to protect cortical and striatal neurons, reduce motor deficits and extend the lifespan in the animal model of HD [158], it is still not fully understood how Nrf2 impacts the formation and aggregation of mtHtt. Tsvetkov et al. [159] have reported that the transfection of striatal neurons with Nrf2 increased the survival of neurons by shortening the mtHtt half-life and accelerating mtHtt clearance. Similarly, Saito et al., when using RS9, a triterpenoid Nrf2 activator, found that Nrf2 induced the expression of microtubule-associated protein 1A/1B-light chain 3 (LC3) and p62 autophagy-related proteins [160] that form a shell surrounding the aggregates of mtHtt [161], thus facilitating their clearance and reducing the toxicity of mtHtt.

Therefore, research on new compounds modulating the activity of Nrf2, which can be used for the treatment of HD, is of great importance. In transgenic mice with HD, the dietary administration of natural triterpenoids resulted in reduced oxidative stress, improved motor impairment, and increased longevity in HD mice [158]. Spectroscopic studies indicate that the activation of Nrf2 can result from the direct binding of triterpenoids with Keap1 at a 2:1 stoichiometry ratio [162] which reduces the availability of Keap1 for Nrf2. Furthermore, naringin, a flavanone from grapefruit, and protopanaxtriol, a compound isolated from ginseng, were reported to decrease the levels of hydroxyl radicals, hydroperoxide and nitrite, as well as inflammatory markers, such as TNFα, COX-2 and iNOS, in the striatum [163]. In addition, these compounds alleviated body weight loss and ameliorated behavior disorder in a 3-Nitropropionic acid animal model of HD [164]. Recently, Moretti et al. [165] designed and synthesized a novel non-acidic naphthalene-derived compound that reversibly binds Keap1 and activates Nrf2. As a result, antioxidant enzyme expression is induced. Importantly, this compound, in contrast to SFN, did not downregulate the expression of genes related to HD, and was not neurotoxic.

Besides the pathological deposition of mtHtt, microglia and astrocyte activation also contributes to the pathogenesis and progression of HD [166], and also chronically elevates pro-inflammatory mediators [167] in other neurodegenerative diseases [168,169]. A bi-directional interplay between the Nrf2 and NF-κB pathways has been extensively documented in a wide range of cell types [170]. Therefore, the activation of Nrf2 decreases the expression of pro-inflammatory enzymes [171] and reduces inflammatory mediator levels, likely via the upregulation of HO-1, which is rapidly induced by oxidative/electrophilic impact [172]. Since HD is accompanied by peripheral inflammation and glial cell activation, the Nrf2/ARE pathway participates in the resolution of neuroinflammation evoked by mtHtt accumulation in neurons. Indeed, it has been reported that the activation of Nrf2 by triazole-containing compounds reduced the IL-6, IL-1β, TNFα and chemokine CCL2 levels in microglia, astrocytes and cortical neurons from HD mice, and in the monocytes of patients with HD [173]. Therefore, it is not surprising that triazole derivatives that suppress NF-κB signaling [174] may activate the Nrf2 pathway and be one of the strategies for HD therapy.

### 5.4. Amyotrophic Lateral Sclerosis

Amyotrophic lateral sclerosis (ALS) is a progressive degenerative disorder caused by a loss of motor neurons in the motor cortex, brain stem, and spinal cord, which subsequently leads to progressive muscle weakness and the loss of voluntary movement control. About 90% of all ALS cases are sporadic (sALS), while the remaining 10% are classified as the familial (fALS) form of the disease. Genetic studies have identified a number of mutations causing familial, as well as sporadic, ALS [175]; among these, the most common are mutations of the following genes: *C9orf72* (chromosome 9 open reading frame 72), *SOD1* (superoxide dismutase 1), *TARDBP* (transactive response (TAR) DNA binding protein 43 kDa) and *FUS* (Fused in Sarcoma (FUS) RNA binding protein), responsible for 40%, 20%, ~6% and ~5% of fALS, respectively [176]. In addition, oxidative stress, neuroinflammation and mitochondrial dysfunction have also been linked to ALS pathogenesis [177].

Similar to the described neurodegenerative diseases, reduced neuronal Nrf2 mRNA and protein expression [178], as well as elevated levels of oxidatively damaged proteins and lipids in the brain and spinal cord of ALS patients [179], was also observed. It is suggested that the expression of nuclear ribonucleoprotein K (hnRNP K) is impaired in TAR DNA binding protein 43 kDa (TDP-43) mutant cells. This RNA binding protein modulates the process of gene expression, including mRNA splicing, export and translation. Hence, a reduction in hnRNP K levels leads to insufficient antioxidant response and motor neuron degeneration, due to the impaired expression of Nrf2-target transcripts [175,180]. Although many studies indicate a reduction of Nrf2 level in ALS, particularly in motor neurons [178,181,182], Kraft et al. [183] reported an increased Nrf2 level in the skeletal muscle of a mouse model of ALS at a very early time point, even before the onset of motor disability. This finding may support the hypothesis that motor neuron degeneration is initiated at the level of neuromuscular junctions [184].

Riluzole, a glutamate antagonist, was the only drug since 1995 for ALS treatment, until 2017, when a new drug, Edaravone, was approved by the Food and Drug Administration (FDA). Edaravone’s neuroprotective activity is based on its ability to partially suppress oxidative stress through the Nrf2/ARE pathway [185,186,187]. Recently, a phase-2 CENTAUR clinical trial showed that a combination of two compounds—sodium phenylbutyrate and tauroursodeoxycholic acid, known as AMX0035—slowed functional decline and significantly extended the life of ALS patients. Both substances increase the expression of Parkinson disease protein 7 (DJ-1/PARK7) [188,189], which stabilizes Nrf2 by preventing its association with Keap1, and subsequent Nrf2 ubiquitination [190]. However, new compounds capable of modulating the Nrf2/ARE pathway are constantly being sought. In animal models of ALS, promising results were obtained with triterpenoids (e.g., CDDO ethylamide and CDDO trifluoroethylamide) and acylaminoimidazole derivatives [191,192]. In turn, Kanno et al., using a novel ligand-based virtual screening system, identified a small molecule, CPN-9, that is able to activate the translocation of Nrf2 to the nucleus in neuronal cells and suppress oxidative stress-induced cell death through the induction of antioxidant enzymes (HO-1, NQO1 and GCLM) [193]. Transgenic ALS mice treated with CPN-9 showed improved motor functions and delayed disease progression. Furthermore, the greater survival of ALS mice has been demonstrated after treatment with triterpenoids that stimulate the translocation of Nrf2 to the nucleus of motor neurons and upregulates Nrf2/ARE-dependent antioxidant gene expression [191]. However, it should be noted that both the type of the pharmacological inducer and the type of targeted cell may be important in finding successful Nrf2-mediated therapy for ALS. This is because, in the astrocytes of transgenic ALS mice, Nrf2 overexpression significantly extended the time of survival, delayed the disease onset and reduced the rate of motor neuron damage [194], while the upregulation of Nrf2 in neurons or in type II skeletal muscle fibers delayed the disease onset but failed to extend survival [195].

## 6. Conclusions

This review provides a brief overview of the Nrf2/ARE-driven transcriptional coordinated cellular responses to stress conditions that play a pivotal role in maintaining the adequate function of neurons and glial cells in the brain. Therefore, the failure of the Nrf2/ARE pathway, along with neuroinflammation and the collapse of mitochondrial function, is one of the reasons contributing to the pathogenesis and progression of neurodegenerative diseases such as AD, PD, ALS and HD. Collectively, the clinical and experimental studies discussed in the present paper evidence an increase in oxidative stress markers and attenuated antioxidant/redox enzyme activity in most neurological disorders. However, Nrf2-driven cytoprotection is multi-directional and multi-level and, to date, it is not clear which point is primarily disrupted in the Nrf2-coordinated enzymatic response to harmful aggregates in the brain. Is it a failure of Keap1-dependent and Keap1-independent Nrf2 activation in the cytoplasm, or the faulty translocation of Nrf2 to the nucleus, or the shortening of Nrf2 half-life, or finally, the incorrect interaction of Nrf2 with other transcription factors during the formation of the transcription preinitiation complex? Furthermore, a consensus has not been achieved regarding whether oxidative stress and mitochondrial dysfunction result from intracellular and extracellular pathological deposits, or whether increased ROS levels and defects in mitochondrial bioenergetics are primary pathogenic factors in neurodegeneration processes. Notwithstanding this, many natural and synthetic compounds that target Nrf2/ARE signaling can be employed for the delay of neurodegenerative disease onset, and even in the therapy of brain disorders. In this context, we consider that Nrf2/ARE pathway activation could be an important target to decrease oxidative damage of brain tissue and to reduce cognitive impairment in patients with neurodegenerative disorders.

## Figures and Tables

**Table 1:** List of *NFE2L2* transcript variants and encoded protein isoforms.

mRNA				Protein			
**Transcript Variant**	**NCBI Reference Sequence**	No of Base Pairs	Information	Isoform	NCBI Reference Sequence	No of Amino Acids	Information
1	NM_006164.5	2446 bp	The longest transcript variant encoding the longest isoform.	1	NP_006155.2	605 aa	The isoform has the canonical sequence.
2	NM_001145412.3	2988 bp	Uses an alternate promoter, 5’ UTR and a downstream start codon vs. var. 1. It has a shorter N-terminus than isoform 1.	2	NP_001138884.1	589 aa	Protein lacks the Keap1 interaction domain, resulting in Nrf2 stabilization. Found in the lung and head-and-neck cancers.
3	NM_001145413.3	2967 bp	Uses an alternate promoter, 5’ UTR, downstream start codon, and an alternate in-frame splice site in the 3’ coding region vs. var. 1. It has a shorter N-terminus and is missing an internal segment than. isoform 1.	3	NP_001138885.1	582 aa	Protein lacks the Keap1 interaction domain, resulting in Nrf2 stabilization.
4	NM_001313900.1	2862 bp	Uses an alternate promoter, 5’ UTR and a downstream start codon vs. var. 1. It has a shorter N-terminus than isoform 1.	2	NP_001138884.1	589 aa	Protein lacks the Keap1 interaction domain, resulting in Nrf2 stabilization. Found in the lung and head-and-neck cancers.
5	NM_001313901.1	2954 bp	Uses an alternate promoter, 5’ UTR and a downstream start codon vs. var. 1. It has a shorter N-terminus than isoform 1.	2	NP_001138884.1	589 aa	Protein lacks the Keap1 interaction domain, resulting in Nrf2 stabilization. Found in the lung and head-and-neck cancers.
6	NM_001313902.1	2769 bp	Lacks an alternate in-frame exon in the 3’ coding region vs. var. 1. It is shorter than isoform 1.	4	NP_001300831.1	575 aa	Computationally mapped isoform.
7	NM_001313903.1	2640 bp	Uses an alternate in-frame splice site in the 3’ coding region vs. var. 1. It is shorter than isoform 1.	5	NP_001300832.1	532 aa	Computationally mapped isoform.
8	NM_001313904.1	2917 bp	Uses an alternate promoter, 5’ UTR and an alternate in-frame splice site in the 3’ coding region, vs. variant 1. It is shorter than isoform 1.	6	NP_001300833.1	505 aa	Computationally mapped isoform.
